# Efficacy of Anti-VEGF therapy for different OCT Patterns in diabetic macular edema and the correlation between ellipsoid zone integrity and visual acuity

**DOI:** 10.1038/s41598-026-47416-7

**Published:** 2026-04-03

**Authors:** Xiao-nan Shi, Qian-ying Zhang, Chao-juan Ju, Yin-cong Xu, Zhao-hui Xiong

**Affiliations:** Department of Ophthalmology, The First Hospital of Hebei Medical University, Shijiazhuang, 050000 Hebei China

**Keywords:** Diabetic macular edema, OCT, Ellipsoid zone integrity, Diseases, Endocrinology, Medical research

## Abstract

To observe the efficacy of Anti-VEGF therapy for different patterns of optical coherence tomography of diabetic macular edema, and to explore the relationship between the integrity of the ellipsoid zone (EZ) and visual acuity outcomes. A total of 90 affected eyes with DME who received anti-VEGF therapy were enrolled in this study. The examination of BCVA was according to Early Treatment Diabetic Retinopathy Study, and the results were recorded as logarithm of the minimum angle of resolution (logMAR). Spectral-domain OCT was used to measure central macular thickness (CMT) and assess the integrity of the ellipsoid zone.All eyes were classified into three groups based on OCT patterns: diffuse retinal thickening (DRT group, 29 eyes), cystoid macular edema (CME group, 30 eyes), and serous retinal detachment (SRD group, 31 eyes). All patients received an intravitreal injection of 0.05 ml (2.5 mg) conbercept.Changes in BCVA and CMT among the three groups were compared and analyzed at baseline and at 3, 6, and 12 months after treatment. The length of the EZ defect was measured. Based on the extent of EZ disruption, eyes were classified into three grades (0, 1, and 2) to analyze the correlation between EZ integrity and visual acuity. In addition, factors influencing post-treatment visual acuity were analyzed. Compared with the central macular thickness (CMT) before treatment, the CMT of the affected eyes was significantly decreased in the diabetic retinal thickening (DRT) group (t = 6.506, 4.134, 7.782, *P* < 0.05), cystoid macular edema (CME) group (t = 15.407, 20.746, 24.749, *P* < 0.05) and serous retinal detachment (SRD) group (t = 9.580, 14.823, 16.931, *P* < 0.05), with statistically significant differences. Compared with the baseline, the best-corrected visual acuity (BCVA) of the affected eyes was significantly improved at 3, 6 and 12 months after treatment in the DRT group (t = 8.667, 17.050, 9.114, *P* < 0.05), CME group (t = 12.104, 11.583, 9.204, *P* < 0.05) and SRD group (t = 7.726, 9.310, 11.757, *P* < 0.05), with statistically significant differences. There were statistically significant differences in the LogMAR BCVA among the DRT, CME and SRD groups before treatment and at each time point after treatment (F = 11.379, 11.461, 15.282, 7.782, *P* < 0.05). A statistically significant difference was observed in the ellipsoid zone grading among the DRT, CME and SRD groups (χ²=21.179, *P* < 0.005). Compared with the baseline, the visual acuity was significantly improved at 3, 6 and 12 months after treatment in patients with ellipsoid zone grade 0 (t = 10.178, 12.375, 8.881, *P* < 0.05), grade 1 (t = 12.353, 13.914, 12.175, *P* < 0.05) and grade 2 (t = 5.154, 8.192, 8.712, *P* < 0.05) of LogMAR BCVA ellipsoid zone grading, respectively. Among the three ellipsoid zone groups, patients with ellipsoid zone grade 0 had the best visual acuity, while those with grade 2 had the worst visual acuity; statistically significant differences were found among the three groups before treatment and at 3, 6 and 12 months after treatment. The visual acuity at 12 months after treatment was significantly correlated with diabetic macular edema (DME) classification, ellipsoid zone defect grading, BCVA before treatment and CMT before treatment (*r* = 0.372, 0.547, 0.745, 0.309, all *P* < 0.05), while no significant correlations were found with age, duration of diabetes, glycosylated hemoglobin A1c (HbA1c), diabetic retinopathy (DR) stage and CMT at 12 months after treatment (*r*=-0.103, -0.035, 0.173, -0.026, 0.084, all *P* > 0.05). Anti-VEGF therapy could significantly improve the visual acuity of different patterns of DME, reduced the CMT, and had the best efficacy in the DRT group. There was relationship between the integrity of ellipsoid zone and the visual acuity outcomes.

**Supplementary Information:**

The online version contains supplementary material available at 10.1038/s41598-026-47416-7.

## Introduction

 Diabetic macular edema (DME), a prevalent complication of diabetic retinopathy (DR), is characterized by the pathological accumulation of fluid within the intraretinal or subretinal spaces of the macula, often leading to profound central vision loss^1–3^. The underlying pathophysiology is multifactorial, involving retinal capillary basement membrane thickening, disruption of endothelial cell tight junctions, pericyte loss, breakdown of the blood-retinal barrier, capillary occlusion, and subsequent neovascularization^4,5^. Given that vascular endothelial growth factor (VEGF) is a key mediator of the hypoxia-induced vascular permeability and proliferation central to these processes^6^, intravitreal anti-VEGF therapy has been established as the first-line treatment for DME^7^.

In recent years, with the development of optical coherence tomography (OCT) imaging technology, in vivo histopathological understanding of DME has continued to deepen. Based on OCT imaging features, DME can be classified into three subtypes: diffuse retinal thickening(DRT), cystoid macular edema (CME), and serous retinal detachment (SRD)^8^. Each subtype has distinct pathophysiological mechanisms, and different DME subtypes exhibit varying responses to treatment, leading to controversy regarding clinical efficacy^9,10^.

In some DME patients, visual acuity may not fully recover after anti-VEGF therapy despite a reduction in macular thickness. This incomplete recovery is likely attributable to damage to the retinal structure, particularly the photoreceptor layer. In recent studies of other fundus diseases such as retinal vein occlusion^11^, age-related macular degeneration^12^, and idiopathic epiretinal membrane^13^, the continuity of the photoreceptor layer has been found to correlate with visual acuity. However, the pathogenesis of DME is more complex, involving multiple factors that influence visual acuity.

This study compared and observed the best-corrected visual acuity (BCVA) of eyes with different DME subtypes after anti-VEGF therapy. It also evaluated the integrity of the ellipsoid zone in DME patients using spectral-domain OCT (SD-OCT) and analyzed its correlation with visual acuity.

### Subjects and Methods

This study was conducted in accordance with the Declaration of Helsinki and approved by the Ethics Committee of the First Hospital of Hebei Medical University (Protocol number: 20200646). Written informed consent was obtained from all patients and their family members after they were informed of the study details. This was a prospective study.

A total of 64 patients (90 eyes) diagnosed with diabetic macular edema (DME) at the First Hospital of Hebei Medical University from January 2021 to December 2024 were enrolled in this study. Among them, there were 30 male patients (45 eyes) and 34 female patients (45 eyes), with ages ranging from 36 to 78 years and a mean age of (56.64 ± 9.799) years.

#### Inclusion criteria

1. Meeting the diagnostic criteria for type 2 diabetes mellitus^14^;
2. Meeting the diagnostic criteria for diabetic macular edema (DME)^15^;
3. Patients with type 2 diabetes receiving standardized treatment and achieving well-controlled blood glucose (fasting blood glucose ≤ 6.1 mmol·L⁻¹, postprandial blood glucose ≤ 11.1 mmol·L⁻¹).

#### Exclusion criteria

1. Refractive error exceeding ± 3.0 diopters (D);
2. Opaque refractive media precluding the acquisition of clear images;
3. Abnormal intraocular pressure (IOP)(IOP > 21 mmHg or < 10 mmHg);
4. Comorbidity with other retinal and choroidal diseases, such as age-related macular degeneration (AMD), retinal vein occlusion (RVO), polypoidal choroidal vasculopathy (PCV), uveitis, cataract surgery and others;
5. History of focal and/or grid pattern retinal laser photocoagulation, vitrectomy, intravitreal injection, or other ophthalmic surgeries;
6. Systemic diseases, including poorly controlled hypertension and abnormal hormone levels, were recorded and considered in the analysis of potential confounding factors.

All patients underwent a comprehensive ophthalmic examination. This included assessments of best-corrected visual acuity (BCVA), intraocular pressure (IOP), slit-lamp biomicroscopy, indirect ophthalmoscopy, and optical coherence tomography (OCT). BCVA was measured using the Early Treatment Diabetic Retinopathy Study (ETDRS) chart, and the results were recorded as the logarithm of the minimum angle of resolution (logMAR).The central macular thickness (CMT)—defined as the retinal thickness from the internal limiting membrane (ILM) to the outer edge of the hyperreflective line of the retinal pigment epithelium (RPE) layer at the fovea centralis of the affected eye—was measured using the Cirrus HD-OCT 5000 device (Carl Zeiss AG, Germany)^16^. All measurements were performed by the same experienced ophthalmologist, with each measurement repeated three times, and the average value was taken for analysis. The integrity of the ellipsoid zone (EZ) reflective band within 500 μm of the fovea along the horizontal foveal scan line was observed, and the length of EZ defect was measured. According to the degree of EZ disruption, the EZ was classified into three grades^17^: Grade 0, intact and continuous EZ; Grade 1, EZ defect length ≤ 200 μm; Grade 2, EZ defect length > 200 μm. To minimize errors in EZ disruption grading due to scan location, we implemented the following measures: (1) During the experiment, we standardized the OCT scan location, focusing on the foveal center and taking 5 consecutive scans around the fovea (100 μm apart from each other) for each subject; (2) For each of the 5 scans, two independent observers measured the EZ disruption grade three times, and the average value was taken as the final grade of the scan; (3) The average grade of the 5 scans around the fovea was used as the final EZ disruption grade of the subject. All enrolled patients underwent optical coherence tomography angiography (OCTA) examination to clearly assess the macular vascular perfusion status. Based on the OCTA findings, we excluded cases with clinically confirmed macular ischemic lesions.

According to the OCT findings, DME was classified into three subtypes: diffuse retinal thickening (DRT), cystoid macular edema (CME), and serous retinal detachment (SRD)^18^. The diagnostic criteria for each subtype were as follows: DRT was defined as diffuse hyporeflectivity within the retina caused by spongiform edema involving an area of ≥ 2 disc diameters (DD) in the macular region; CME was characterized by hyporeflective cystic spaces of varying sizes separated by hyperreflective septa within the macular retina; SRD was identified as separation between the neurosensory retina and the retinal pigment epithelium (RPE) in the macular region.

DRT was defined as diffuse hyporeflectivity solely due to spongiform edema. For the purpose of grouping, any eye presenting with CME, either alone or in combination with DRT, was assigned to the CME group. Similarly, any eye with SRD, regardless of the presence of DRT or CME, was assigned to the SRD group.Based on the above classification criteria, the patients were divided into three groups: the DRT group, the CME group, and the SRD group.

Glycated hemoglobin (HbA1c) levels were collected from all patients, and the stage of diabetic retinopathy (DR) was recorded. DR was classified into two stages: (1) Non-proliferative Diabetic Retinopathy (NPDR); (2) Proliferative Diabetic Retinopathy (PDR).

Treatment protocol: All intravitreal injections were administered in a sterile laminar flow operating room in accordance with standard sterile surgical protocols. A 1‑ml syringe was used to draw up 0.05 ml of conbercept (2.5 mg), which was injected transconjunctivally at the pars plana, 3.5–4.0 mm posterior to the limbus in the inferotemporal quadrant.

A 3 + PRN treatment regimen was applied. This consisted of three consecutive monthly loading doses, followed by additional injections as needed, administered by the same physician based on clinical evaluation.

Retreatment criteria were as follows: (1) A decrease of 5 letters in best-corrected visual acuity (BCVA); (2) An increase of > 100 μm in central macular thickness (CMT). Injection termination criteria were as follows: (1) BCVA of the affected eye > 84 letters in two consecutive re-examinations; (2) Stable visual acuity in three consecutive re-examinations without further improvement after at least two consecutive injections.

The follow-up period after treatment was ≥ 12 months. At 3, 6, and 12 months after treatment, the same equipment and methods as those before treatment were used to perform relevant examinations to observe the changes in BCVA and CMT of the affected eyes and the occurrence of treatment-related complications.

All data were analyzed using SPSS 27.0 for Windows (SPSS Inc., Chicago, IL, USA). A value of *P* < 0.05 was considered statistically significant.

1. Handling of bilateral eye data and adjustment of confounding factors: Considering that some patients had bilateral eyes included in this prospective study, inter-eye correlation was considered a potential confounding factor. Generalized Estimating Equations (GEE) were applied to analyze the data to account for the correlation between paired eyes from the same patient, thus avoiding treating fellow eyes as independent observations. Specifically, GEE models were used to adjust for potential confounding factors including baseline HbA1c (given significant differences among the three DME subgroups) and inter-eye correlation when assessing treatment outcomes across subgroups. For analyses that did not involve bilateral eye data (e.g., comparisons of count data, proportions of PDR and NPDR, differences in EZ grading among groups), inter-eye correlation was not applicable and thus not considered.
2. Description of data expression and group comparison methods: Measurement data were expressed as mean ± SD (± s). For comparisons of measurement data between groups, analysis of variance (ANOVA) or t-test was used as appropriate; for count data, which were expressed as frequency and composition ratio [N(%)], χ² test was used for group comparisons. Fisher’s exact test was specifically used for comparing the proportions of PDR and NPDR and the differences in ellipsoid zone (EZ) grading among each group.
3. Analysis of changes in indicators at different time points: Repeated measures analysis of variance (ANOVA) was used to analyze the changes of indicators (including BCVA and CMT) at different follow-up time points within each DME subtype group. When significant differences were found by repeated measures ANOVA, the least significant difference (LSD) t-test was used for pairwise comparison of differences at each time point. For analyses involving bilateral eye data (e.g., changes in BCVA and CMT), GEE models were simultaneously used to account for inter-eye correlation.
4. Correlation analysis methods: The relationships between 12‑month post‑treatment BCVA and clinical parameters were initially explored using correlation analysis, with methods selected based on the type of variables to ensure appropriateness. For correlations between two continuous variables (e.g., correlation between baseline CMT and 12-month BCVA), Pearson correlation analysis was used, and Pearson correlation coefficients (r) and corresponding p‑values were reported. For correlations involving ordinal variables (including DME subtypes and ellipsoid zone (EZ) grades) with continuous variables (e.g., correlation between EZ grading and BCVA), Spearman’s rank correlation analysis was used, as these ordinal variables do not meet the application conditions of Pearson correlation analysis.

## Results

There were 20 patients (29 eyes) in the DRT group, accounting for 32.22%; 24 patients (30 eyes) in the CME group, accounting for 33.33%; and 20 patients (31 eyes) in the SRD group, accounting for 34.44%. Comparisons of age, duration of diabetes, HbA1c, number of injections, and DR stage among the three groups are shown in Table 1.

**Table 1:** Baseline demographic and clinical characteristics of patients by DME subtype.

Group	Number of Eyes	Age ($\:\stackrel{-}{\mathrm{x}}$±s, years)	Duration of Diabetes ($\:\stackrel{-}{\mathrm{x}}$±s, years)	HbA1c ($\:\stackrel{-}{\mathrm{x}}$±s, %)	Number of Injections ($\:\stackrel{-}{\mathrm{x}}$±s)	Number and Proportion of PDR [*n*(%)]	Number and Proportion of NPDR [*n*(%)]
DRT group	29	55.17 ± 10.10	9.03 ± 2.65	7.50 ± 0.55	3.52 ± 1.184	13 (44.8%)	16 (55.2%)
CME group	30	55.60 ± 10.49	8.20 ± 3.26	8.06 ± 0.94	3.53 ± 1.008	13 (43.3%)	17 (56.7%)
SRD group	31	59.03 ± 8.61	8.77 ± 2.54	9.13 ± 1.30	3.84 ± 1.186	17 (54.8%)	14 (45.2%)
Test Value	-	F = 1.432	F = 0.673	F = 21.326	F = 0.784	χ²=0.972	-
P	-	0.244	0.513	< 0.001	0.46	0.649	-

Note: There was a significant difference in HbA1c levels among the three groups (F = 21.326, *P* < 0.001), with the HbA1c levels in the CME group and SRD group being significantly higher than those in the DRT group (t=−3.004, −6.516, all *P* < 0.05). No significant differences were observed in age, duration of diabetes, number of injections, or DR stage among the three groups (all *P* > 0.05).Measurement data were expressed as mean ± SD, and analyzed by ANOVA or t-test; count data were expressed as [N(%)], and analyzed by χ² test; inter-eye correlation was not considered in these analyses.

**Fig. 1:**
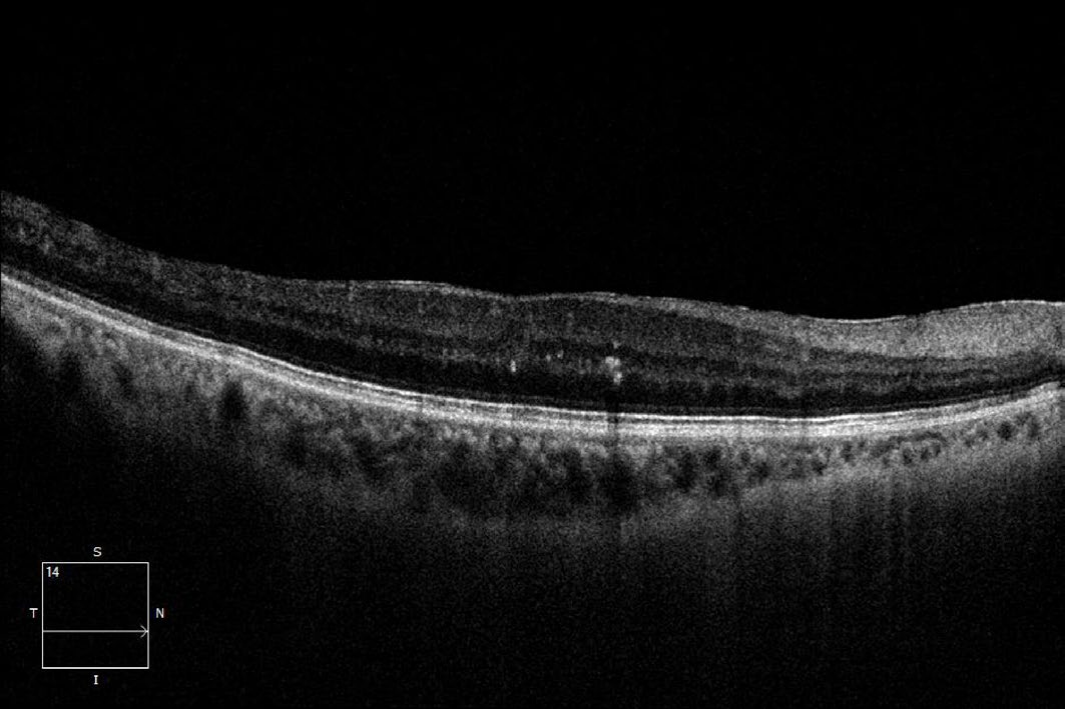
DRT-type macular edema.

**Fig. 2:**
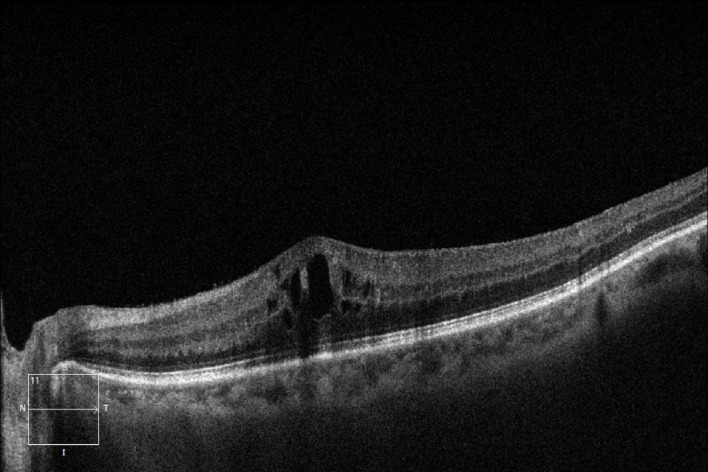
CME-type macular edema.

**Fig. 3:**
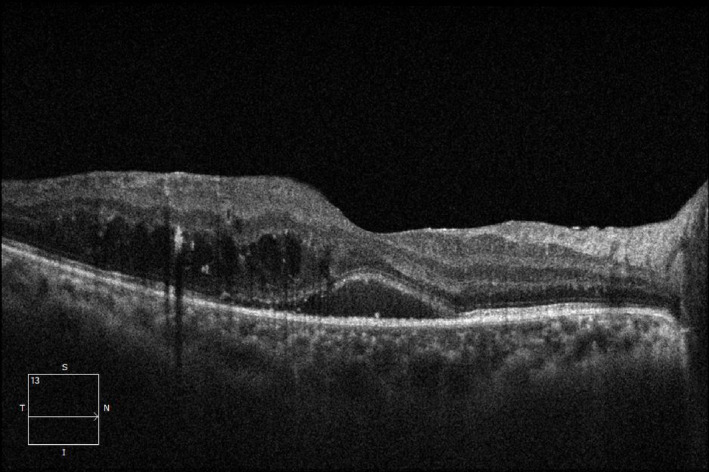
SRD-type macular edema.

After adjustment for baseline HbA1c imbalance, differences in treatment response among DME subtypes remained statistically significant (Fig. 1, 2, 3).

In the DRT group, the number and proportion of eyes with ellipsoid zone (EZ) grade 0 were the highest, accounting for 17 eyes (58.6%). In the CME group, the number and proportion of eyes with EZ grade 1 were the highest, accounting for 16 eyes (53.3%). In the SRD group, the proportions of eyes with EZ grade 1 and grade 2 were the highest, accounting for 14 eyes (45.2%) and 13 eyes (41.9%), respectively. There was a statistically significant difference in EZ grading among the DRT group, CME group, and SRD group (χ²=21.179, *P* < 0.005), as shown in Table 2.

**Table 2:** Distribution of ellipsoid zone (EZ) Grade by DME subtype.

Group	EZ Grade 0 [*n*(%)]	EZ Grade 1 [*n*(%)]	EZ Grade 2 [*n*(%)]
DRT group	17 (58.6%)	11 (37.9%)	1 (3.4%)
CME group	10 (33.3%)	16 (53.3%)	4 (13.3%)
SRD group	4 (12.9%)	14 (45.2%)	13 (41.9%)
χ²	21.179		
P	< 0.001		

**Fig. 4:**
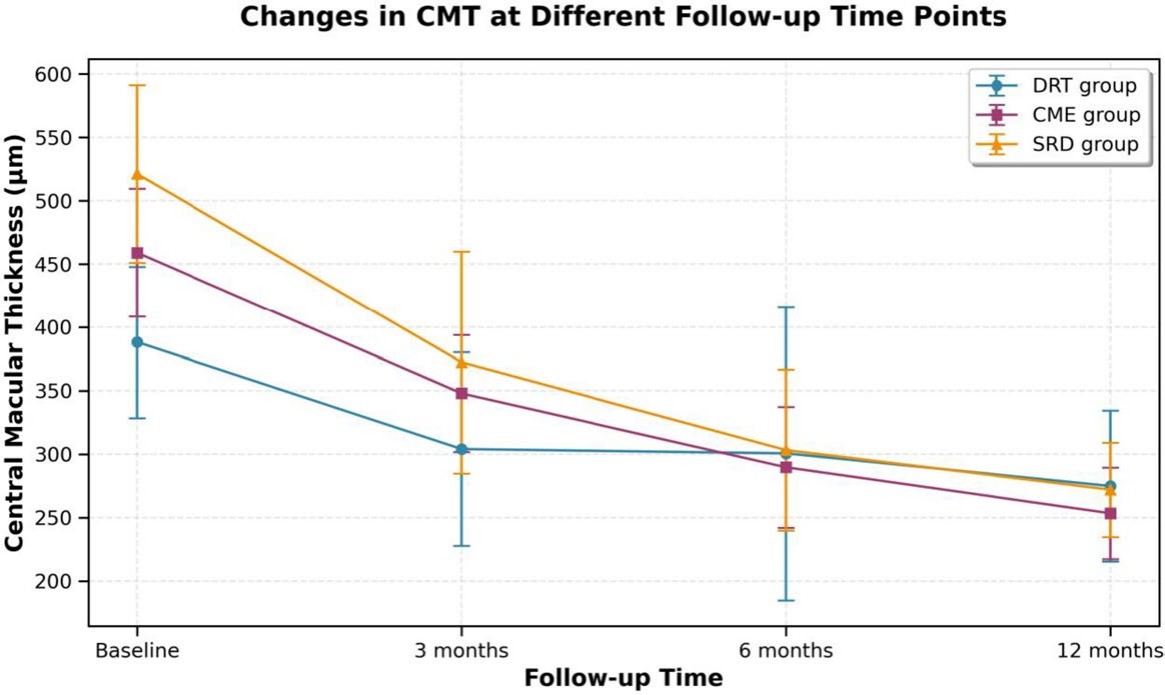
Changes in CMT Values of Patients in the DRT Group, CME Group, and SRD Group Before and After Treatment.

Changes in BCVA and CMT at different follow-up time points within each DME subtype group were analyzed by repeated measures ANOVA, with LSD t-test for pairwise comparisons; GEE models were used to account for inter-eye correlation in these analyses (*P* < 0.05 for significant differences).Before treatment and at 3, 6, and 12 months after treatment, the central macular thickness (CMT) values of the DRT group were (388.10 ± 59.76), (304.00 ± 76.21), (300.62 ± 115.76), and (274.86 ± 59.49) µm, respectively; those of the CME group were (459.10 ± 50.58), (347.80 ± 45.92), (289.63 ± 47.64), and (253.37 ± 36.18) µm, respectively; and those of the SRD group were (521.29 ± 70.10), (372.29 ± 87.61), (303.19 ± 63.28), and (272.00 ± 37.19) µm, respectively, as shown in Table 3.Compared with the CMT before treatment, the CMT of the affected eyes in all three groups was significantly decreased, with statistically significant differences: DRT group (t = 6.506, 4.134, 7.782, all *P* < 0.05), CME group (t = 15.407, 20.746, 24.749, all *P* < 0.05), and SRD group (t = 9.580, 14.823, 16.931, all *P* < 0.05).Statistically significant differences in CMT were observed among the three groups at baseline (F = 35.961, *P* < 0.05) and at 3 months post-treatment (F = 6.833, *P* < 0.05).No statistically significant differences in CMT values were observed among the three groups at 6 and 12 months after treatment (F = 0.243, 1.982; *P* = 0.785, 0.144), as shown in Fig. 4.

**Table 3:** Changes in Central Macular Thickness (CMT) at different time points in each group.

Group	CMT (baseline)	CMT (3 months)	CMT (6 months)	CMT (12 months)	F	*P*
DRT group	388.10 ± 59.76	304.00 ± 76.21	300.62 ± 115.76	274.86 ± 59.49	18.93	<0.001
CME group	459.10 ± 50.58	347.80 ± 45.92	289.63 ± 47.64	253.37 ± 36.18	259.09	<0.001
SRD group	521.29 ± 70.10	372.29 ± 87.61	303.19 ± 63.28	272.00 ± 37.19	135.04	<0.001

**Fig. 5:**
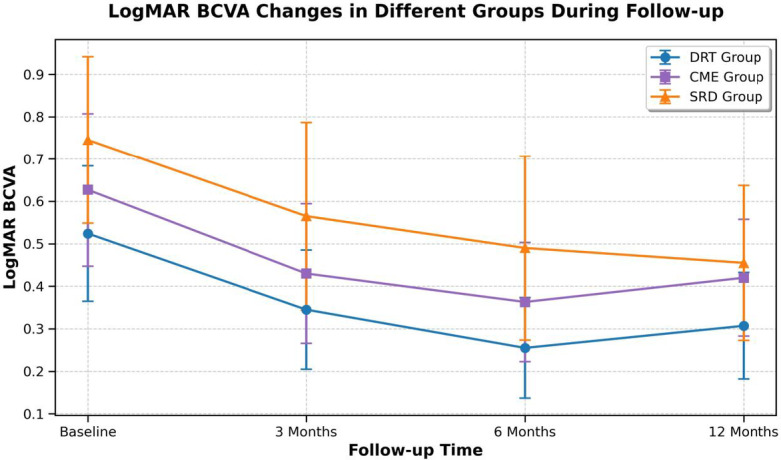
Changes in logMAR BCVA values of patients in the DRT group, CME group, and SRD group before and after treatment.

Before treatment and at 3, 6, and 12 months after treatment, the logarithm of the minimum angle of resolution best-corrected visual acuity (logMAR BCVA) values of the DRT group were 0.524 ± 0.1596, 0.345 ± 0.1404, 0.255 ± 0.1183, and 0.307 ± 0.1252, respectively; those of the CME group were 0.627 ± 0.1799, 0.430 ± 0.1643, 0.363 ± 0.1402, and 0.420 ± 0.1375, respectively; and those of the SRD group were 0.745 ± 0.1964, 0.565 ± 0.2214, 0.565 ± 0.2214, and 0.455 ± 0.1823, respectively, as shown in Table 4.Compared with the logMAR BCVA before treatment, the BCVA of the affected eyes in all three groups was significantly improved at 3, 6, and 12 months after treatment, with statistically significant differences: DRT group (t = 8.667, 17.050, 9.114, all *P* < 0.05), CME group (t = 12.104, 11.583, 9.204, all *P* < 0.05), and SRD group (t = 7.726, 9.310, 11.757, all *P* < 0.05).There were statistically significant differences in logMAR BCVA values among the DRT group, CME group, and SRD group before treatment and at each time point after treatment (F = 11.379, 11.461, 15.282, 7.782, all *P* < 0.05), as shown in Fig. 5.

**Table 4:** Changes in LogMAR best-corrected visual acuity (LogMAR BCVA) at different time points in each group.

Group	LogMAR BCVA (baseline)	LogMAR BCVA (3 months)	LogMAR BCVA (6 months)	LogMAR BCVA (12 months)	F	*P*
DRT group	0.524 ± 0.1596	0.345 ± 0.1404	0.255 ± 0.1183	0.307 ± 0.1252	73.154	<0.001
CME group	0.627 ± 0.1799	0.430 ± 0.1643	0.363 ± 0.1402	0.420 ± 0.1375	79.002	<0.001
SRD group	0.745 ± 0.1964	0.565 ± 0.2214	0.490 ± 0.2166	0.455 ± 0.1823	57.424	<0.001

Note: Statistical analysis was performed by repeated measures ANOVA with LSD t-test for pairwise comparisons; GEE models were used to account for inter-eye correlation (*P* < 0.05 was considered statistically significant).

Among all DME patients, 31 eyes (34.3%) were classified as ellipsoid zone (EZ) grade 0, 41 eyes (45.6%) as EZ grade 1, and 18 eyes (20.0%) as EZ grade 2. Comparisons of logMAR BCVA before treatment and at each time point after treatment among different EZ grade groups are presented in Table 5.

**Table 5:** Changes in visual acuity before and after treatment among different ellipsoid zone (EZ) grade groups.

Group	Number of Eyes and Proportion [*n*(%)]	logMAR BCVA (baseline)	logMAR BCVA (3 months)	logMAR BCVA (6 months)	logMAR BCVA (12 months)
EZ Grade 0	31 (34.3%)	0.503 ± 0.1581	0.342 ± 0.1409	0.261 ± 0.1086	0.313 ± 0.1204
EZ Grade 1	41 (45.6%)	0.639 ± 0.1656	0.427 ± 0.1644	0.356 ± 0.1550	0.380 ± 0.1249
EZ Grade 2	18 (20.0%)	0.850 ± 0.1383	0.683 ± 0.1724	0.600 ± 0.1815	0.572 ± 0.1708
F	-	27.455	27.204	30.772	21.896
P	-	< 0.001	< 0.001	< 0.001	< 0.001

Compared with those before treatment, the logMAR BCVA was significantly improved in all three EZ grade groups at 3, 6, and 12 months after treatment, with statistically significant differences: EZ grade 0 group (t = 10.178, 12.375, 8.881, all *P* < 0.05), EZ grade 1 group (t = 12.353, 13.914, 12.175, all *P* < 0.05), and EZ grade 2 group (t = 5.154, 8.192, 8.712, all *P* < 0.05).

**Fig. 6:**
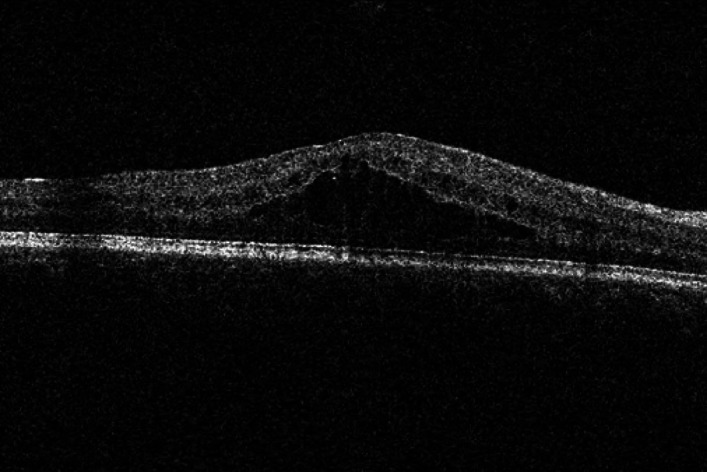
Ellipsoid Zone (EZ) Grade 0.

**Fig. 7:**
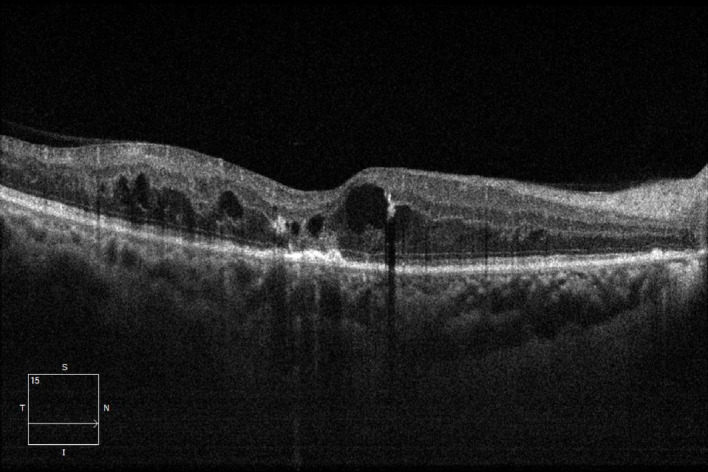
Ellipsoid Zone (EZ) Grade 1.

**Fig. 8:**
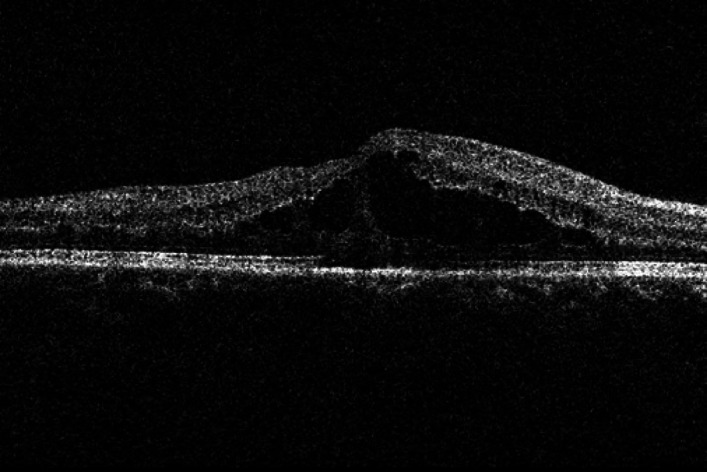
Ellipsoid Zone (EZ) Grade 2.

Among the three EZ grade groups, the EZ grade 0 group had the best visual acuity, while the EZ grade 2 group had the worst visual acuity. There were statistically significant differences in visual acuity among the three groups before treatment and at 3, 6, and 12 months after treatment (F = 27.455, 27.204, 30.722, 21.896, all *P* < 0.05) (Fig. 6, 7, 8).

**Fig. 9:**
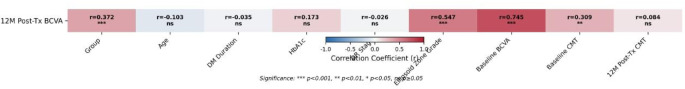
Correlation of 12-month post-treatment BCVA with clinical variables. Note Heatmap of correlation coefficients between 12-month post-treatment BCVA and various clinical parameters.The heatmap was generated using Python 3.9.13 with Matplotlib 3.7.1 and Seaborn 0.12.2 (https://www.python.org/, https://matplotlib.org/, https://seaborn.pydata.org/). * indicates statistical significance (*P* < 0.05).

Visual acuity at 12 months after treatment was significantly correlated with DME subtypes, ellipsoid zone defect grading, BCVA before treatment, and CMT before treatment (*r* = 0.372, 0.547, 0.745, 0.309, all *P* < 0.05). It was not correlated with age, duration of diabetes, HbA1c, DR stage, or CMT at 12 months after treatment (*r*=−0.103, −0.035, 0.173, −0.026, 0.084, all *P* > 0.05),as shown in Fig. 9.

## Discussion

Our study demonstrated that Anti-VEGF Therapy improved BCVA and CMT across different DME subtypes, and EZ disruption was associated with worse visual outcomes. To avoid bias caused by inter-eye correlation in this bilateral eye-included prospective study, GEE models were used to account for inter-eye correlation and adjust for baseline HbA1c imbalance when analyzing treatment outcomes; meanwhile, correlation analysis methods were selected based on variable types (Pearson correlation for continuous variables, Spearman’s rank correlation for ordinal variables), ensuring the reliability and clarity of statistical results. These findings are consistent with previous studies and provide new insights into the treatment of DME subtypes.

This prospective study investigated the efficacy of anti-VEGF therapy (conbercept) in three distinct OCT-based subtypes of DME and evaluated the correlation between baseline ellipsoid zone (EZ) integrity and visual acuity outcomes. Our main findings are threefold. First, anti-VEGF treatment significantly improved BCVA and reduced CMT in all DME subtypes over the 12-month follow-up. Second, the therapeutic response varied among subtypes.Although DRT subtype tended to show favorable visual improvement in this study, baseline HbA1c was significantly higher in the CME and SRD groups, which may confound treatment outcomes. After adjustment for this imbalance, the observed differences remained, suggesting that baseline glycemic control does not fully account for the subtype-dependent response to conbercept.Third, baseline EZ integrity was strongly correlated with visual outcomes, serving as a significant prognostic indicator. Although DME is characterized by the accumulation of fluid in and/or under the neurosensory retina of the macular area, leading to increased retinal thickness, it can be clinically classified into three types based on OCT morphological features: DRT, CME, and SRD^19^. Studies have shown that these three types differ in their pathogenesis and prognosis. DRT type is mainly related to the breakdown of the inner blood-retinal barrier and increased retinal vascular permeability caused by inflammation and oxidative stress^20^; the intraretinal cystoid changes in CME type may be derived from the liquefaction or apoptosis of Müller cells and the action of inflammatory cytokines such as prostaglandins^21^; while SRD type is associated with retinal pigment epithelium (RPE) dysfunction, which leads to increased choroidal capillary permeability, allowing exudate to accumulate in the subretinal space through the RPE layer and the outer limiting membrane^22^.

In this study, after anti-VEGF treatment, the visual acuity of patients in the DRT group, CME group, and SRD group was significantly improved compared with that before treatment. Among them, the baseline visual acuity and post-treatment visual acuity of the DRT group were better than those of the other two groups, which was consistent with the results of previous studies^23^. DRT, often considered an early morphological change in DME, is characterized by the absence of cystoid spaces or subretinal fluid on OCT. VEGF plays a major role in its pathogenesis^24^; VEGF is a multifunctional growth factor that causes extracellular fluid accumulation by disrupting the tight junctions of retinal vascular endothelial cells and increasing vascular permeability^25^. Conbercept has a high affinity with VEGF, which inhibits VEGF from binding to its receptors, thereby decreasing vascular leakage and reducing macular edema^26^. Therefore, DRT typically shows a favorable anatomical and functional response to anti-VEGF therapy, leading to a relatively good visual prognosis.

On the basis of DRT, CME will have liquefaction and necrosis of Müller cells leading to cystoid changes. Its pathogenesis is not only related to VEGF but also closely linked to inflammatory cytokines^27^. SRD type may be related to persistent inflammation and ischemia-hypoxia of the body, which leads to deformation of the retinal pigment epithelium layer structure, disorder of the retinal pigment barrier function, and accumulation of fluid in the subretinal space, resulting in macular lesions^28^. Therefore, simple anti-VEGF treatment may have poor efficacy for CME-type and SRD-type macular edema. In recent years, more and more studies have shown that for CME-type and SRD-type macular edema, the application of anti-VEGF combined with intravitreal dexamethasone implant can achieve a good visual prognosis^29,30^.

It should be noted that there was no significant difference in the number of injections among the three groups, but there was a significant difference in HbA1c levels. The HbA1c level in the SRD group was significantly higher than that in the CME group and DRT group, which also reflects that the severity of macular edema is closely related to systemic blood glucose status^31,32^.

SD-OCT can not only accurately measure the degree of macular edema in patients with DME but also measure and analyze the outer retinal structure. The outer retinal structure is mainly composed of photoreceptors, which are displayed as four light bands from the inside out on SD-OCT images: the outer limiting membrane, myoid band, ellipsoid zone (EZ), and outer segments of photoreceptors. Our analysis revealed a significant correlation between the extent of EZ disruption and visual acuity. Specifically, greater EZ defect lengths were associated with poorer BCVA at all time points, suggesting that there is a correlation between the integrity of EZ and visual acuity. Pearson correlation analysis also confirmed that the length of EZ defect was strongly correlated with visual acuity, which was consistent with the results of previous studies^33–35^.The lower severity of EZ disruption in the DRT group may be related to the pathological characteristics of different macular lesions: (1) DRT is mainly characterized by diffuse retinal thickening without obvious accumulation of intraretinal or subretinal fluid, so the EZ structure is less likely to be directly damaged or masked; (2) In the CME group and SRD group, the presence of intraretinal edema or subretinal fluid may mask the EZ structure, leading to an overestimation of the severity of EZ disruption, or the fluid itself may directly damage the EZ layer, resulting in more obvious disruption.

Among the three groups (DRT group, CME group, and SRD group), the proportions of patients with EZ destruction degree of grade 1 and grade 2 were 41.3%, 66.6%, and 87.1%, respectively. With the aggravation of DME severity, the degree of EZ destruction also gradually increased. At 6 and 12 months after treatment, there was no statistically significant difference in CMT values among the three groups, but there was a statistically significant difference in visual acuity among the three groups. This indicates that although CMT is related to BCVA, it cannot be used alone to predict BCVA prognosis^36,37^. A study by Jithin et al. showed that when CMT > 300 μm, the continuity of EZ has little effect on visual acuity; when CMT < 300 μm, the continuity of EZ has a great effect on visual acuity. Therefore, whether the EZ is continuous can be used as an indicator to predict the visual prognosis of patients, but not as an indicator to decide whether to treat. When patients have macular edema, we should still give them active treatment.

With the continuous deepening of our understanding of the pathophysiological effects of DME on the retina, based on the research results on the structure and function of retinal structures such as the EZ, we may be able to more reasonably apply existing therapies and design more targeted and effective future treatment regimens for DME.

### Strengths and limitations

The strengths of this study include its prospective design, relatively large sample size, and 12-month follow-up period, which allowed for a robust longitudinal assessment. The use of standardized OCT protocols and double-observer grading enhanced the reliability of our measurements. However, several limitations must be acknowledged. First, we did not longitudinally track changes in EZ disruption over time, which could provide insights into the reversibility of photoreceptor damage. Second, despite our efforts, EZ grading remains a semi-quantitative method with potential for subjective bias. Third, we did not control for all potential confounding factors, such as concomitant medications or the duration of DME prior to treatment. Baseline HbA1c differed significantly among subgroups, which was adjusted in statistical analysis but may still exert residual confounding effects. Finally, the molecular mechanisms linking different DME subtypes to EZ damage were not explored and warrant further investigation. Future studies should focus on these aspects to validate and expand upon our findings.Date availability statement: All data supporting the conclusions of this study are included as additional files in the supplementary information associated with this article and are available upon request from the corresponding author.

## Supplementary Information

Below is the link to the electronic supplementary material.

Supplementary Material 1

## Data Availability

All data supporting the conclusions of this study are included as additional files in the supplementary information associated with this article and are available upon request from the corresponding author.
